# Advanced characterization of biomineralization at plaque layer and inside rice roots amended with iron- and silica-enhanced biochar

**DOI:** 10.1038/s41598-020-80377-z

**Published:** 2021-01-08

**Authors:** Guanhong Chen, Sarasadat Taherymoosavi, Soshan Cheong, Yao Yin, Rabeya Akter, Christopher E. Marjo, Anne M. Rich, David R. G. Mitchell, Xiaorong Fan, Jinkiat Chew, Genxing Pan, Lianqing Li, Rongjun Bian, Joseph Horvat, Mohanad Mohammed, Paul Munroe, Stephen Joseph

**Affiliations:** 1 National-Regional Joint Engineering Research Center for Soil Pollution Control and Remediation in South China, Guangdong Key Laboratory of Integrated Agro-Environmental Pollution Control and Management, Institute of Eco-Environmental and Soil Sciences, Guangdong Academy of Sciences, Guangzhou, 510650 China; 2 School of Materials Science and Engineering, University of New South Wales, Sydney, NSW 2052 Australia; 3 Mark Wainwright Analytical Centre, University of New South Wales, Sydney, NSW 2052 Australia; 4 Electron Microscopy Centre, AIIM Building, Innovation Campus, University of Wollongong, North Wollongong, NSW 2517 Australia; 5 College of Resources and Environmental Sciences, Nanjing Agricultural University, Nanjing, 210095 China; 6 Institute for Superconducting and Electronic Materials and School of Physics, University of Wollongong, Wollongong, NSW 2522 Australia

**Keywords:** Environmental sciences, Biogeochemistry

## Abstract

Application of iron (Fe)- and silica (Si)-enhanced biochar compound fertilisers (BCF) stimulates rice yield by increasing plant uptake of mineral nutrients. With alterations of the nutrient status in roots, element homeostasis (e.g., Fe) in the biochar-treated rice root was related to the formation of biominerals on the plaque layer and in the cortex of roots. However, the in situ characteristics of formed biominerals at the micron and sub-micron scale remain unknown. In this study, rice seedlings (*Oryza sativa L.*) were grown in paddy soil treated with BCF and conventional fertilizer, respectively, for 30 days. The biochar-induced changes in nutrient accumulation in roots, and the elemental composition, distribution and speciation of the biomineral composites formed in the biochar-treated roots at the micron and sub-micron scale, were investigated by a range of techniques. Results of laser ablation inductively coupled plasma mass spectrometry (LA-ICP-MS) showed that biochar treatment significantly increased concentrations of nutrients (e.g., Fe, Si, and P) inside the root. Raman mapping and vibrating sample magnetometry identified biochar particles and magnetic Fe nanoparticles associated with the roots. With Fe plaque formation, higher concentrations of FeO_x_^−^ and FeO_x_H^−^ anions on the root surface than the interior were detected by time-of-flight secondary ionization mass spectrometry (ToF-SIMS). Analysis of data from scanning electron microscopy energy-dispersive spectroscopy (SEM-EDS), and from scanning transmission electron microscopy (STEM) coupled with EDS or energy electron loss spectroscopy (EELS), determined that Fe(III) oxide nanoparticles were accumulated in the crystalline fraction of the plaque and were co-localized with Si and P on the root surface. Iron-rich nanoparticles (Fe–Si nanocomposites with mixed oxidation states of Fe and ferritin) in the root cortex were identified by using aberration-corrected STEM and in situ EELS analysis, confirming the biomineralization and storage of Fe in the rice root. The findings from this study highlight that the deposition of Fe-rich nanocomposites occurs with contrasting chemical speciation in the Fe plaque and cortex of the rice root. This provides an improved understanding of the element homeostasis in rice with biochar-mineral fertilization.

## Introduction

Rice (*Oryza sativa* L.), which serves as the dietary staple for half the world’s population, has experienced increasing demand with global population growth^1^. Rice is normally cultivated in flooded paddy soil, and takes up essential and beneficial mineral elements for growth^2^. Under anaerobic conditions, with high levels of iron (II) (Fe^2+^), Fe homeostasis plays an essential role in many cellular functions of the plant, and can provide protection from toxicity of free-form Fe as catalyst of the Fenton reaction^3^. Fe-rich plaque was formed around the rice root by rhizosphere oxygenation following radial oxygen loss through root aerenchyma to the root surface and oxidation of Fe(II) at the root surface (i.e., biologically-induced biomineralization)^4^. Further, rice roots can take up Fe^3+^ by phytosiderophore, and Fe^2+^ followed by Fe chelation and reduction^5^. To store Fe in a safe and bioavailabile form, the iron oxide nanoparticles (such as ferrihydrite (5Fe_2_O_3_·9H_2_O) in ferritin) are formed in the cytocol and organelles of the root (i.e., matrix-mediated biomineralization)^6^.

Biochar amendments, such as wheat straw-derived biochar, have been reported to increase rice yield through improving the physical and biochemical properties of the soil and have been widely applied in paddy fields^7–9^. The biochars produced from wheat straw, one of the most abundant crop residues^10^, are enriched with silicon (Si) and thereby have a beneficial effects on plants^11^. It has been reported that biochars prepared through slow pyrolysis enhanced plant growth by their liming effect and high nutrient availability^12,13^. Combining Fe-rich minerals (e.g., FeSO_4_ and Fe_2_O_3_), together with chemical compounds containing nitrogen (N) and phosphorus (P), biochar-based fertilizers produced at relatively low temperature (350–450 °C) through slow pyrolysis possessed a high concentration of carbon (C)-containing functional groups (e.g., carboxylic C) and dissolved organic matter, which was allowed to be more bioavailable to soil microbes and was higher redox active in the rhizosphere^14^. Previous research has shown that application of Fe-enhanced biochar can promote the formation of Fe-rich plaque and increase Fe accumulation in rice plants, and alter the uptake, transportation and accumulation of other mineral nutrients (e.g., N and P) as well as toxic trace elements (e.g., Cd and As) in rice plants^15–17^. Our previous study revealed that the Fe- and Si-enhanced biochar enable to induce higher rice yield and N and P use efficiency relative to conventional chemical fertilizer, which was attributed to the greater plant uptake of mineral nutrients via the increased soil Eh and plant-growth promoting bacteria^15^. With response to the alteration of mineral nutrient status in root, element homeostasis (e.g., Fe) of rice root has been often analyzed by molecular genetic approaches^18^. Despite the importance of biomineralization in regulating cytoplasmic free mineral ions in plant, there is limited research on the in situ characteristics of biominerals formed on the plaque layer and in the cortex in the biochar-treated rice roots at the micron and sub-micron scale.

Microscopic and spectroscopic techniques enable spatial distribution, concentration, and chemical speciation on mineral elements in plant tissues to be determined. Scanning electron microscopy coupled with energy-dispersive spectroscopy (SEM-EDS) was the traditional method to detect the composition of macronutrients that accumulate to high concentration within plant tissues^19^. Energy-dispersive X-ray microanalysis (EDS) of the interface between a wheat root and Fe-enriched biochar particle showed that high concentrations of Fe, Mn, Si, and Ca, and significant concentrations of P, Mg, Al, and K were present^20^. The same approach was used to characterise the Fe biominerals in *Imperata cylindrica*, an Fe hyperaccumulator, and this study showed crystalline Fe-containing phases formed in the root xylem vessels^21^. Transmission electron microscopy (TEM) coupled with EDS or electron energy-loss spectroscopy (EELS) analyses can be used to determine the chemical elements present and their speciation in plant tissues with high spatial resolution and sensitivity, such as a copper complex bound to a cell wall^22^, and Fe-bearing particles in the organelles and vacuoles of root cells^23^. TEM has been used to show the subcellular location of nanoparticles in plant root tissue exposed to Fe oxide nanoparticles^24^. TEM-EDS analysis of root section of *Imperata cylindrica* showed Fe crystalline deposits with a high concentration of Fe in the root cell wall^25^. Advanced scanning transmission electron microscopy (STEM), coupled with energy electron loss spectroscopy (EELS), has been used to demonstrate the phase structure and oxidative states of nanoparticles in biological tissues^26^. Advanced STEM-EDS/EELS analysis has the capability to characterise the composition and speciation of biominerals at near nanometer scale within plant root tissue.

An imaging mass spectrometry approach, time-of-flight secondary ionization mass spectrometry (ToF-SIMS), has been utilized to verify the elemental and molecular distribution within plant tissues^27^. ToF-SIMS analysis revealed the distribution of elements such as Si, Al, Fe, Ca, Mg, Na, and K at the interface of tree roots with soil^28^, metals ions in wood tissue sections^29^, and coniferin distributions in ginkgo stems^27^. Another mass spectrometry-based technique is laser ablation inductively coupled plasma mass spectrometry (LA-ICP-MS) which can provide semiquantitative trace analysis of essential and toxic elements in plant tissues^30^. LA-ICP-MS analysis has been used to detect Cu and Zn in the surface tissues of cucumber root^31^.

The objective of this study is to utilize all these complementary characterization techniques to identify biochar-induced effects on nutrient accumulation in roots and the concomitantly formed Fe- and Si-rich biominerals in, or on, the rice root treated with the Fe- and Si-enhanced wheat straw biochar. The in situ composition, distribution and speciation of biominerals within plaque layers and inside the cortex of rice roots at micron- and sub-micron scale were studied using SEM-EDS, STEM-EDS/EELS, ToF-SIMS, LA-ICP-MS, and atomic force microscopy (AFM), as well as vibrating sample magnetometry (VSM) and Raman spectroscopy.

## Results

### Elemental concentration at the exterior and interior surfaces of root in the control or biochar-amended soil

**Figure 1:**
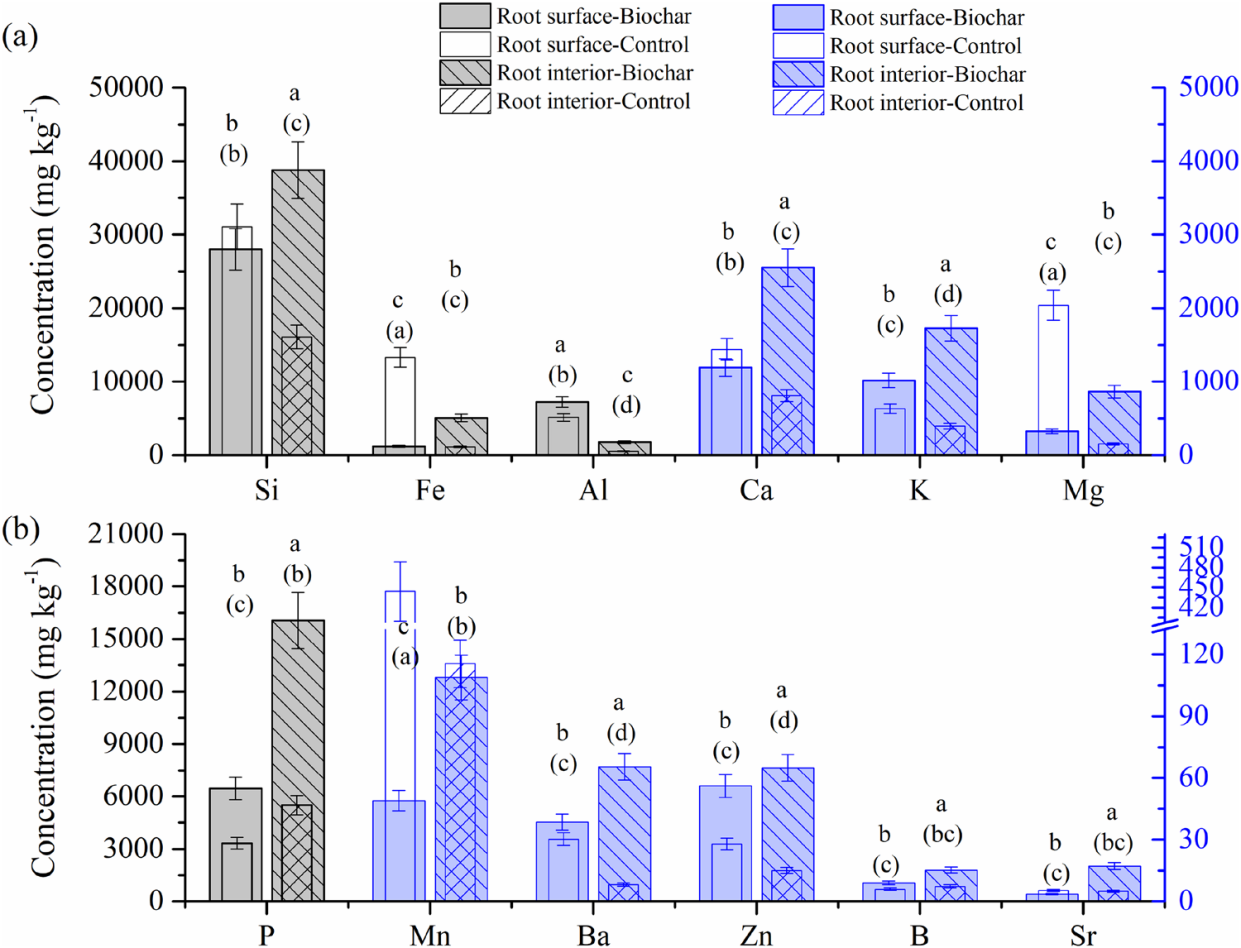
Total elemental concentrations (derived from LA-ICP-MS) of (**a**) Si, Fe, Al, Ca, K, and Mg and (**b**) P, Mn, Ba, Zn, B, and Sr at the surface and interior of the rice root in the control and biochar treatment. Error bars show standard deviation of three repeated measurements. Different lowercase letters indicate significant difference between the control (in brackets) and biochar treatment for each element (*P* < 0.05).

With biochar addition, the mineral elements at the root interior were significantly higher than the surface, except for Al. At the root interior, there were significantly higher average concentration of nutrients in the biochar-treated root than the control root, such as Fe (5048 vs. 1120 mg kg^−1^ dry weight), Si (38,781 vs*.* 16,103 mg kg^−1^ dry weight), and P (16,050 vs*.* 5495 mg kg^−1^ dry weight) (Fig. 1). The concentrations of Ca, K, Mg, B, Zn, and Al were also found to increase significantly inside the biochar-treated root (cf. control), while the Mn was not affected by biochar addition. To reveal the characteristics of biominerals formed in rice root containing high Fe and Si concentrations following biochar treatment, the elemental composition and speciation at the surface and interior of biochar-treated root were detected at the micron and sub-micron scale.

### Elemental composition at the exterior and interior surfaces of the biochar-treated rice root

A magnetic hysteresis loop of a root sample is shown in Fig. S1a. Magnetic field strength was applied in the range of − 5000 to 5000 Oe. The S-shaped curve with saturation magnetization value of 0.052 emu g^−1^ at 5000 Oe showed weak superparamagnetic behavior of the roots. The coercive field of the root was 58 Oe. Figure S1b shows a Raman spectrum from dark aggregates attached to the root surface. Two overlapping Raman bands at 1350 cm^−1^ (D band) and 1580 cm^−1^ (G band) were assigned to the in-plane vibrations of a sp^2^-bonded carbon structure, which is characteristic of amorphous char structure in biochar particles (Fig. S1b)^32^. Raman D band is activated by structural disorder in graphitic structures and it corresponds to breathing modes of carbon sp^2^ rings. The G band corresponds to the in-plane stretching vibrations of C–C bonds in graphitic systems and it does not require structural disorder to become Raman active. Raman mapping of the G band showed that micron-sized biochar particles (originally < 25 μm in size) appeared to form aggregates, ranging in size from < 10 up to 40 μm (Fig. S1c—inset). Dark regions were produced by detector saturation from autofluorescence.

**Figure 2:**
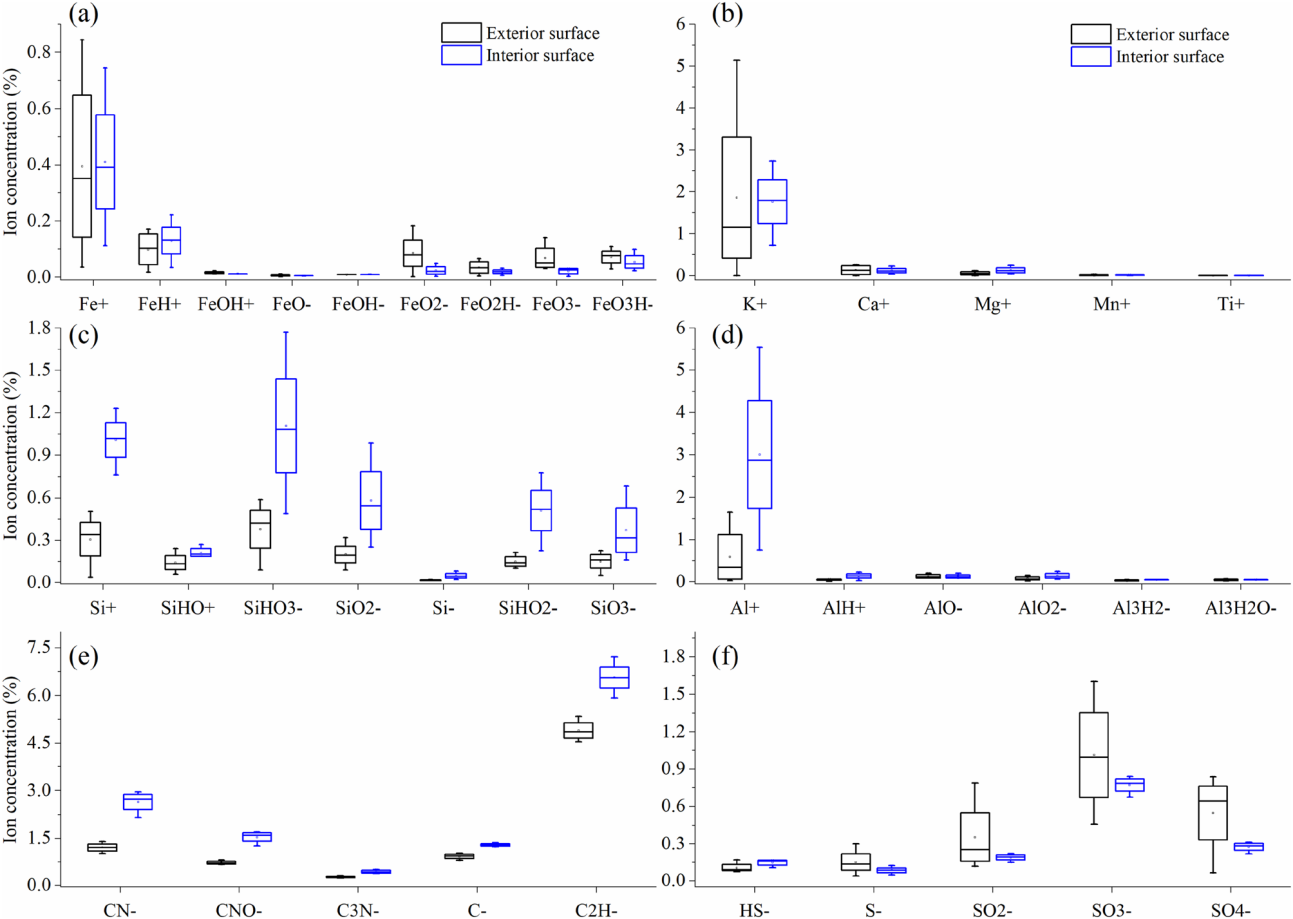
Box plots of relative concentrations of (**a**) Fe-bearing ions, (**b**) K, Ca, Mg, Mn, and Ti cations, (**c**) Si-bearing ions, (**d**) Al-bearing ions, (**e**) C-bearing anions, and (**f**) S-bearing anions, at the exterior and interior surface of the biochar-treated rice root detected by ToF-SIMS. Each plot represents readings from five locations randomly selected in a single specimen.

Elemental compositions in the roots were further characterised using ToF-SIMS measurements in both positive and negative modes. ToF-SIMS revealed that the relative concentrations of FeO_2_^−^, FeO_2_H^−^, FeO_3_^−^, and FeO_3_H^−^ anions (Fig. 2a), as well as S-bearing anions (Fig. 2f) at the interior surface of the root were generally lower in concentration than those at the exterior root surface. However, the ions of Ca, Mg, Si, Al, and C were more abundant at the interior than the exterior (Fig. 2b–e). The most abundant Fe- and Al-bearing ions were Fe^+^ (0.035–0.84%) and Al^+^ (0.030–5.5%). The dominant Si-bearing ion liberated from the root was SiHO_3_^−^ (0.089–1.8%) followed by Si^+^ (0.035–1.2%). While SiHO_3_^−^ and SiHO_2_^−^ anions indicated the presence of hydrated amorphous Si compounds, the Si^+^ cation corresponded to silicates. Anions of C_2_H^−^, CN^−^, and CNO^−^ were derived from organic phases that were abundant in the root. In addition, the sulfur oxide anions, mainly SO_3_^−^ and SO_4_^−^, were also present at both the exterior and interior regions of the root.

**Figure 3:**
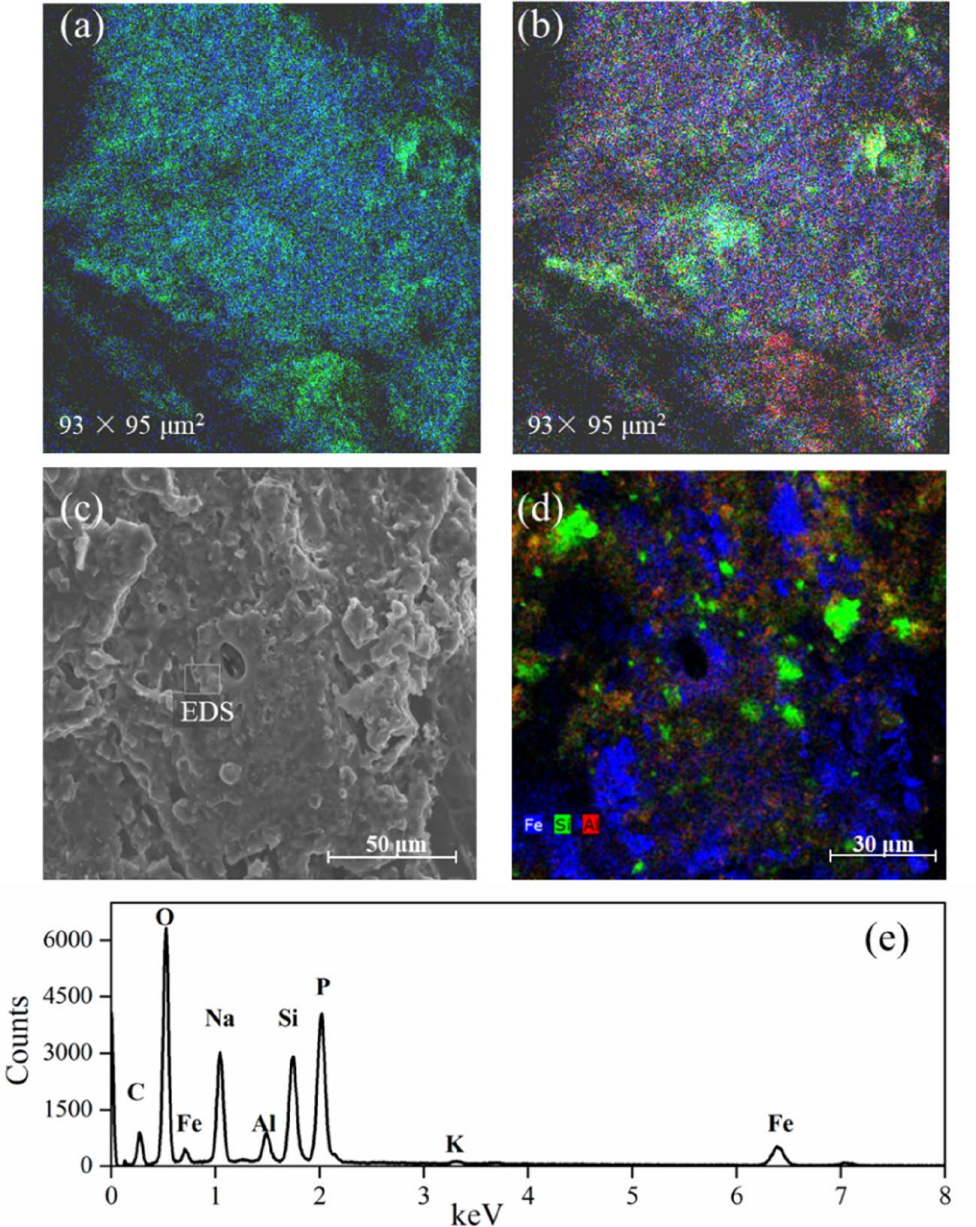
The morphology and elemental distribution at the exterior surface of the biochar-treated rice root. ToF-SIMS images of (**a**) the sum of CN^−^ and CNO^−^ (green) and Fe-bearing ions (Fe^+^, FeH^+^, FeOH^+^, FeO^−^, FeOH^−^, FeO_2_^−^, FeO_2_H^−^, FeO_3_^−^, FeO_3_H^−^) (blue); and (**b**) the sum of Fe-bearing ions (blue), Al-bearing ions (Al^+^, AlH^+^, AlO^−^, AlO_2_^−^, Al_3_H_2_^−^, Al_3_H_2_O^−^) (red) and Si-bearing ions (Si^+^, SiHO^+^, SiO_2_^−^, SiHO_3_^−^, Si^−^, SiHO_2_^−^, SiO_3_^−^) (green). (**c**) SEM secondary electron image of the exterior surface of the root and (**d**) EDS mapping showing the elemental distribution of Fe, Al, and Si, and (**e**) EDS spectrum obtained from the region of interest in Fig. 4c, showing strong signals from P, Si, Al and Fe.

**Figure 4:**
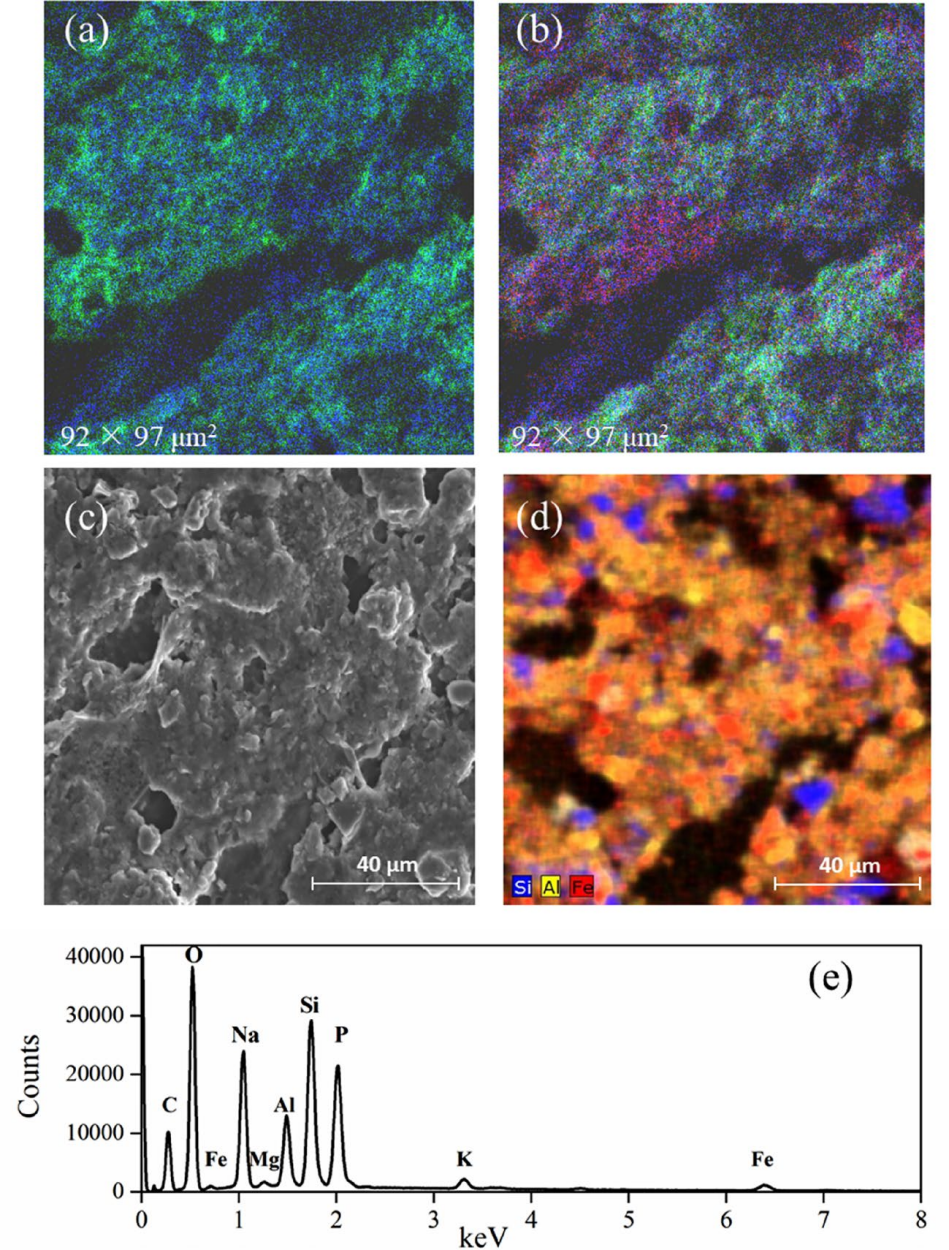
The morphology and elemental distribution at the interior surface of the biochar-treated rice root. ToF-SIMS images of (**a**) the sum of CN^−^ and CNO^−^ (green) and Fe-bearing ions (Fe^+^, FeH^+^, FeOH^+^, FeO^−^, FeOH^−^, FeO_2_^−^, FeO_2_H^−^, FeO_3_^−^, FeO_3_H^−^) (blue); and (**b**) the sum of Fe-bearing ions (blue), Al-bearing ions (Al^+^, AlH^+^, AlO^−^, AlO_2_^−^, Al_3_H_2_^−^, Al_3_H_2_O^−^) (red) and Si-bearing ions (Si^+^, SiHO^+^, SiO_2_^−^, SiHO_3_^−^, Si^−^, SiHO_2_^−^, SiO_3_^−^) (green). (**c**) SEM secondary electron image of the interior surface of the root and (**d**) EDS mapping showing the elemental distribution for Fe, Al, and Si, (**e**) EDS spectrum obtained from the whole region shown in Fig. 5c, showing strong signals from Si, P, Al, and Fe.

ToF-SIMS measures the outermost monolayer of a sample surface to determine micron-scale distributions of elements and molecules. Mapping the intensity of the CN^−^ and CNO^−^ anions produces an image of the protein distribution in the interior and exterior surface of the root (Figs. 3a and 4a). Silicon-bearing ions yielded a strong signal in some regions of the root exterior (Fig. 3b). The spatial distributions of Fe- and Al-bearing ions mainly coincided with the distributions of Si-bearing ions. SEM imaging was undertaken on the same regions as those analysed by ToF-SIMS (Fig. 3c). The analysis revealed that some large organomineral composites were formed on the root surface. EDS mapping showed that regions which were rich in Fe and those rich in Si did not coincide exactly, whereas Al-rich clusters were restricted to either the Fe- or Si-rich regions (Fig. 3d). Elemental concentrations analyzed by EDS spectroscopy were ranked in order: P > Si > Al > Fe (Fig. 3e), although more precise quantification of such a matrix was not feasible. Based on the results of SEM-EDS, Fe-containing aggregates on the root exterior ranged in size from a few to over 100 μm in diameter, indicative of Fe-rich plaque present on the root surface. At the interior surface of the root, the ToF-SIMS analysis showed that Fe-, Al- and Si-bearing ions correlated with the presence of CN^−^ and CNO^−^ (Fig. 4a,b). SEM-EDS results showed that Fe appeared to be highly localised within regions < 1 μm in size (Fig. 4c,d). High concentrations of Si, P, Na, and Al with significant concentrations of Fe, Mg, K, and S within the root interior were also observed by EDS analysis (Fig. 4e).

### Elemental speciation of extracellular and intracellular components of the biochar-treated rice root

**Figure 5:**
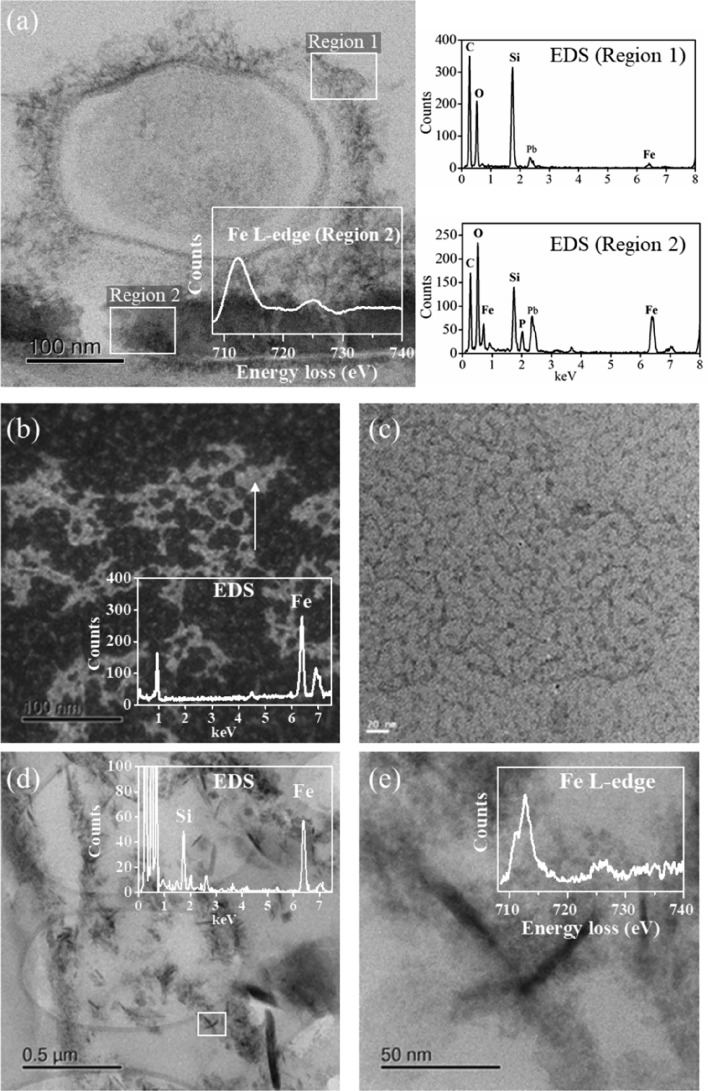
STEM analyses of a transverse section of a rice root exposed to enhanced biochar. (**a**) Bright field (BF) image of a microbe attached to the root epidermis. The two rectangular regions of interest are where EDS/EELS analyses were performed. The EDS spectra show a concentration of Si with small amounts of Fe at the surface layer of the microbe (Region 1), and significant concentrations of Si, Fe, and P at the microbe-root interface (Region 2). The Fe L-edge EEL spectrum of region 2 is shown inset in (**a**) where the edge shape is typical of Fe_3_O_4_. (**b**) High-angle annular dark field (HAADF) image of the intracellular agglomerate of the root. The EDS spectrum from the region arrowed is shown inset where a high concentration of Fe is detected. (**c**) A magnified BF image of the agglomerate showing matrices containing an iron-rich core with a diameter of around 5 nm. (**d**) BF image of an intracellular cluster of particles showing high concentrations of Si and Fe detected by EDS analysis. (**e**) Detailed view of the boxed region in (**d**) showing the needle-like particles, with the inset showing the Fe L-edge EEL spectrum typical for iron minerals with mixed oxidation states (Fe(II)/Fe(III)).

Examination of a stained transverse section of the root by STEM-EDS/EELS revealed the speciation of nanocomposites at the sub-micron scale. Ultrastructural observations (Fig. 5a and Fig. S2) showed that microbes (approximately circular in cross section) with a size of around 0.3 μm were bound to the root epidermis (bottom of the image). Two regions of interest were further analyzed by EDS/EELS. The EDS spectra from Region 1 showed a high concentration of Si with small amounts of Fe at the surface layer of the microbe, and Region 2 showed significant concentrations of Si, Fe, and P at the microbe-root interface. The interface between the microbe and root, a region approximately 100 nm wide (region 2), was analyzed by EELS—the Fe L-edge is shown inset in Fig. 5a. There was a sharp peak at ~ 712.0 eV in the Fe L-edge, suggesting iron in a Fe(III) oxidation state. In the interior of the root, intracellular agglomerates were visualized by high-angle annular dark field (HAADF) imaging (bright regions in Fig. 5b). The EDS spectrum from the region arrowed is shown as an inset where a high concentration of Fe was detected. A magnified bright field (BF) image of the agglomerate showed that matrices contained an Fe-rich core with a diameter of around 5 nm (Fig. 5c). Additionally, there were intracellular particle clusters shown in the BF image (Fig. 5d) and high concentrations of Si and Fe were detected by EDS analysis (Fig. 5d—inset). A detailed view of the clusters showed the presence of needle-like particles (Fig. 5e). The corresponding Fe L-edge EEL spectrum (inset) showed that the Fe L_3_ edge had maximum at ~ 712.8 eV with a split edge typical for Fe minerals with a mixed oxidation states (Fe(II)/Fe(III)) (Fig. 5e—inset).

### Electric potential in the interior of biochar-treated rice root

**Figure 6:**
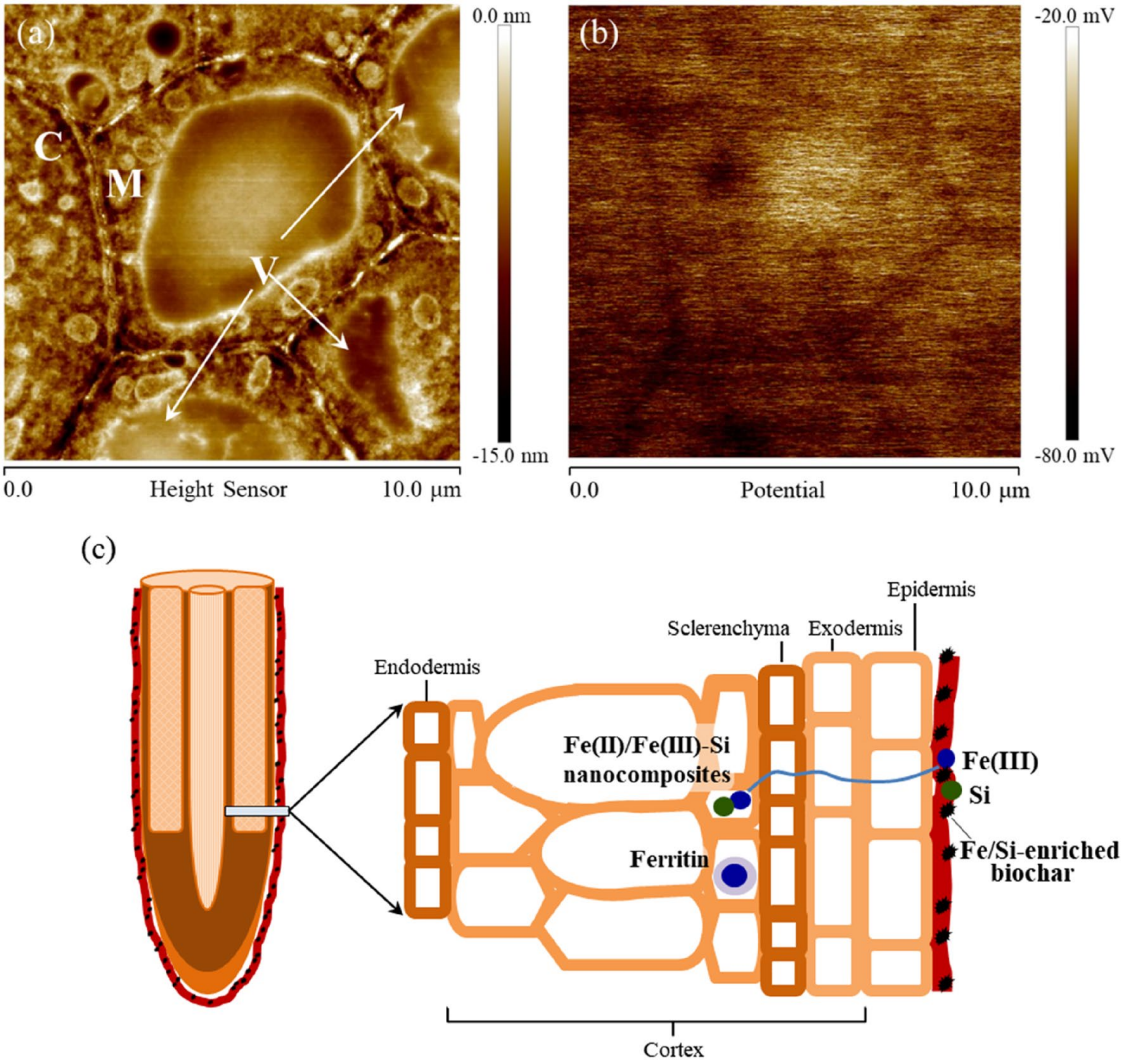
AFM images of a transverse section of a biochar-treated root embedded in resin. (**a**) Topographic image and (**b**) surface potential image. Internal cell structures including a vacuole (V), cytoplasm (C), and mitochondria (M) are apparent. (**c**) Schematic diagram of the formation of a complex Fe/Si phase in iron plaque on the root surface and iron-rich nanocomposites and ferritin within the rice root amended with Fe- and Si-enhanced biochar.

The AFM in Kelvin probe mode (KPFM) visualized the surface topography of cross sections of root tissue to detect the in situ internal cell structure and mapped electrical potential. The AFM image showed the inner ultrastructure of the root, including the cell wall, vacuoles (V), cytoplasm (C), and mitochondria (M) (Fig. 6a). The thickness of the cell wall was approximately 0.2 μm and intracellular structures were readily visible. The thin root section (~ 400 nm) promoted good surface conductivity, improving the resolution of the AFM. The cellular electric potential was detectable as a surface potential (Fig. 6b). It was noted that electrically charged domains presented on a micron scale in the root interior and the potential varied between various internal root structures. The cell wall has a consistently lower surface potential (approximately 20–30 mV) than the cell interior (particularly vacuoles), while a more heterogeneous potential distribution was observed in the cytoplasm.

## Discussion

The increased rice yield by the application of the Fe- and Si-enhanced biochar relative to conventional chemical fertilizer was reported^15^, and this was related to the higher plant uptake of mineral nutrients during the biochar-root interactions^33,34^. The presence of biochar particles on the root surface was identified by Raman spectroscopy. In this spectrum, the observed overlapping D and G band in the range from 1400 to 1550 cm^−1^ (Fig. S1b) is characteristic of the graphitic carbon matrix^32^. Raman mapping of the G band demonstrated that biochar particles were micron-sized (Fig. S1c). Through the direct interaction with biochar, plant roots can take up macro- and micro-nutrients from the surface and from the pores of the biochar^14,15,35^. Significantly higher concentrations of Fe were observed inside the biochar-treated root than the control (Fig. 1), possibly due to additional Fe arising from the biochar, despite the biochar-induced increase in soil pH (from 5.4 to 6.4) counteracting, to some extent, the increased availability of Fe to plants. Compared with the root interior, the average concentrations of iron oxide derived species, i.e., FeO_2_^−^, FeO_2_H^−^, FeO_3_^−^, and FeO_3_H^−^ anions, were greater at the surface of biochar-treated root, as analyzed by ToF-SIMS (Fig. 2), revealing iron oxides and hydroxides formed in the plaque layer^36^. STEM-EELS analysis indicated that the Fe was in the Fe(III) oxidation state. Comparison of the experimental EELS spectrum (Fig. 5a) with a reference EEL spectra for ferrihydrite (5Fe_2_O_3_·9H_2_O) showed a good match^37^. On the other hand, lower concentration of total Fe at root surface was caused by biochar addition (cf. control), likely due to the dilution of Fe in plaque layer with the increased other minerals (e.g., P).

ToF-SIMS measurements showed that SiO_x_^−^ and SO_x_^−^ anions (Fig. 2), ascribed to silicate and sulphate, were located at the root surface. The silicates and sulphates can combine with iron to produce secondary mineral phases in the plaque^38,39^. STEM-EDS determined nanoscale Fe–Si–P association occurred on the root surface, and suggested that in Fe plaque ferrihydrite bears both Si and P at trace levels (Fig. 5a). ToF-SIMS mapping also supported the co-location of Fe- and Si-bearing ions (Fig. 3b). In SEM-EDS analysis, however, the Si and Fe distributions did not completely correlate at the exterior surface of the root (Fig. 3d). These differences may stem from the fact that the EDS signal originates from the outer 1 to 5 microns of the specimen, while ToF-SIMS measures a nanoscale surface monolayer^40^. When wheat straw biochar (400 °C) used in our study was enriched with Si, the Si release from phytogenic Si occurred and could be favored by biochar-induced increase in soil pH^11,41^. The Fe- and Si-enhanced biochar could be considered as a Si source for the Si-bearing ions in the plaque layer, and might have contributed to increased root uptake of Si as analyzed by LA-ICP-MS (Fig. 1). Futhermore, the concentration of P at the root interior and exterior surface was significantly increased by biochar amendment (Fig. 1). Besides the additional P from the biochar, the soil pH, increased by biochar additions, could increase P availability by reduced P sorption from soil (particularly Fe and Al oxides)^42^. Another proposed mechanism was the increase in activity of alkaline phosphatase or phosphodiesterase from microbes, which catalyzed the hydrolysis of esters and anhydrides of phosphoric acid, under liming^43^. With respect to the distribution of Al-bearing ions, Al was associated with the Fe plaque and possibly interacted with Si at the root exterior, as detected by both ToF-SIMS and SEM-EDS. The Al immobilization in the plaque might be due to the formation of aluminosilicate complexes and this could be favored with increasing soil pH through biochar additions^44^. Thus, with the Fe- and Si-enhanced biochar addition, Fe nanocomposites were embedded within the Fe plaque that can sequester and (subsequently) supply plant nutrients of Fe, P, S, and Si, and influence the availability of toxic metals ions (e.g., Al)^45^.

Biochar-rhizosphere interactions can create microbial hotspots, which were defined as small soil volumes with high microbial activity and fast reaction rates, by the labile root exudates and the particulate biochar C^46^. Using STEM, microbes were found to be bound to the Fe-rich plaque in regions with significant concentrations of Fe, Si and P on the biochar-treated root surface (Fig. 5a). The wheat straw biochar used in our study contained a relatively high concentration of low molecular weight water soluble/volatile organic compounds, such as alkenes, alkanes aldehydes, ketones, acids and alcohols, and humic substances and polyphenols^15^. Biochar with redox-active moieties (e.g., (hydro)-quinones,) may act as an electron shuttle from the C substrate to less accessible electron acceptors (e.g., Fe), thus stimulating microbial degradation of organic matter (referred to as the “priming effect”)^33^. The biochar-regulated electron transfer in redox reactions, such as the transformation of C, N and S, can involve reactions with Fe species in the rhizosphere, and this might be one of mechanisms for the significantly increased presence of mineral nutrients in the rice root.

The potential AFM image revealed that the root cell wall had a consistently low potential (Fig. 6b), as it is composed of negatively charged polysaccharides. Silica uptake from biochar-amended soil by a rice root could accumulate in the cell wall, and subsequently form a negatively charged Si-hemicellulose complex which is more likely to bind cations^47^. Cation accumulation in the root cortex could have contributed to micron-sized electrically charged domains in the potential AFM image (Fig. 6b,c). At the root interior, the simultaneous presence of Fe, Al, and Si deposits has been observed by ToF-SIMS (Fig. 4b), indicating the formation of aluminosilicate, which could be responsible for the alleviation of Al toxicity by reducing Al transportation. Despite the observed increase of Al concentration in the biochar-treated root relative to the control (Fig. 1), the coprecipitation of Al with Si is regarded as part of the Al-tolerance strategies in plant^48^.

The nanoparticles < 50 nm from Fe- and Si-enhanced biochar-amended soil could be internalized and translocated through the symplastic pathways, according to the size exclusion limits imposed by chemical and physiological barriers of the root^49^. There have been some recent studies on the morphology of iron oxide nanoparticles within plant roots^24^. Iron in the form of an oxyhydroxide or hydrated oxide species in rice root has been found by Mössbauer spectroscopic measurement of powdered root tissue in a study by Kilcoyne et al.^6^. To our knowledge, this is the first study that has investigated the in situ chemical speciation of intracellular Fe at the nanoscale with aberration-corrected STEM, providing information about the Fe biominerals within the internal structure of a biochar-treated rice root. STEM-EDS/EELS showed that Fe-bearing nanocomposites were present inside the root. These may be ferritin and other Fe-bearing nanoparticles (Fig. 5b,d). The ~ 5 nm Fe core of ferritin was identified by STEM-EDS (Fig. 5b,c). As an Fe-storage protein, ferritin retains Fe in a safe and bioavailable form, providing intracellular control of Fe homeostasis^3^. In addition to ferritin, intracellular needle-like clusters of Fe nanoparticles, with diameters less than 50 nm, which contained significant concentrations of Si were found (Fig. 5d); these may be aegirine (NaFeSi_2_O_6_). EELS of this phase (Fig. 5e) suggested it had a mixed oxidation state (Fe(II)/Fe(III)) in the aegirine phase^50^. The presence of Fe-containing magnetic nanoparticles in the biochar-treated rice root were also supported by VSM, showing a small coercive field (58 Oe) in magnetic hysteresis loop (Fig. 1a) characteristic of magnetic nanoparticles with diameters of less than 50 nm^51^. The Fe-rich core of ferritin and intracellular Fe nanocomposites with mixed oxidation states suggested Fe storage via biomineralization in the biochar-treated rice root^25^. Therefore, with the Fe- and Si-enhanced biochar addition the biominerals were formed in contrasting species at the root exterior and interior, in relation to the mineral homeostasis in rice grown in biochar-fertilizer amended soil.

## Conclusions

A range of advanced techniques were applied to reveal the increase of mineral nutrients and in situ charateristics of concomitantly formed Fe biominerals in rice roots treated with the Fe- and Si-enhanced biochar. LA-ICP-MS showed that the concentrations of mineral nutrients, e.g., Fe, Si, and P, in the root interior increased significantly by biochar treatment. Chemical distributions and speciation of mineral nanocomposites formed in biochar-treated root interior and exterior have been determined at the micron- and sub-micron scale. VSM magnetometry and Raman mapping permitted identification of micron-sized biochar particles and magnetic nanoparticles associated with the roots. ToF-SIMS and SEM-EDS analyses, revealed the formation of a complex Fe–Si–P phase in Fe plaque on the root surface. The sequestration of Al in this phase may reduce the transport of Al. STEM-EDS/EELS showed the presence of Fe-rich nanocomposites and possibly ferritin within the rice root. Additionally, the distribution of subcellular electric potential on a root section can be detected by AFM. These analyses demonstrate the power of such methods to characterize biominerals and provides insights into function of biomineralization in regulating element balance in plants amended with biochar-mineral fertilizer.

## Methods

### Biochar and soil

The Fe- and Si-enhanced biochar fertilizer was produced from a mixture of wheat straw, minerals and nutrients subjected to a relatively low temperature (400 °C), which has been reported elsewhere to increase plant yield compared with conventional fertilizers^15,52^. A suspension of 5 g Fe_2_O_3_, 5 g FeSO_4_·7H_2_O, 15 g rock phosphate, 15 g bentonite clay, and 15 g urea in 100 mL deionized water was prepared. Wheat straw (200 g) was mixed with the suspension at 80 °C and the mixture left to stand for 24 h and then dried at 110 °C for 3 h^53^. The modified biochar was produced by a slow pyrolysis process in a furnace under oxygen-limited conditions with a heating rate of 5 °C min^−1^, held at 400 °C for 30 min, and then cooled to room temperature over a period of 24 h. The detailed properties of this biochar are reported in the study of Chew et al.^15^. The C, N, K, and P composition (wt%) in the biochar are: C 43%; N 2.7%; K 2%; total P 1.4%; citrate-extractable P 1.1%. The pH of the biochar was 6.8, with an acid neutralising capacity of 5.8% in calcium carbonate equivalents. The Fe-enriched bulk biochar included ferromagnetic and superparamagnetic iron oxide nanoparticles with diameters of < 10 nm and > 50 nm, respectively, and sub-micron SiO_2_ particles.

The Fimi-Orthic Anthrosol was collected from the 0 to 15 cm layer from a rice paddy field in Nanjing, China (31° 58′ N, 118° 48′ E). The soil was a clay-loam soil, The soil pH was 5.4, EC 13.5 μS cm^−1^, organic matter was 13.53 mg g^−1^.

### Rice plant growth

The plant growth experiment was carried out in 6.4 L boxes with three replicates. The box was divided into a central compartment with half of the total volume and two side compartments with one quarter of the total volume each. The compartments were separated using double nylon netting with a mesh size of 25 μm. The central compartment contained 2500 g soil, and side compartments were filled with a mixture of 1250 g soil, 12.5 g biochar, and 350 g (NH_4_)_2_SO_4_. Thus, only biochar and mineral particles with diameters of less than 25 μm could migrate to the plant roots. There were biochar treatments (n = 3) and one control (n = 3). The chemical fertilizer was initially added into the soil with final concentrations of N (0.095%), P (0.075%) and K (3.8%), which was indicative of the control. The N, P and K in the biochar treatment (0.095% N, 0.077% P, and 3.8% K) were consistent with the control, as the increase of these macronutrients by biochar was negligible. Rice seedlings (*Oryza sativa L.*) were grown in the central compartment under flooded conditions for 30 days. Plants were placed in a greenhouse with a 14/10 h day/night photoperiod, at 30 °C/22 °C day/night, light intensity of 400 μmol photons m^−2^ s^−1^, and a relative humidity of 65–70%.

### Magnetic properties

Information on sample preparation and characterization techniques of the root sample is given in Table 1, which summarizes the microscopic, spectroscopic and mass spectrometric techniques used. The roots were sampled at harvest on day 30, then washed with deionized water and dried by critical point drying (CPD). Magnetic studies were performed with a vibrating sample magnetometer (VSM) from Quantum Design Physical Property Measurement System. Magnetic hysteresis loops were obtained at room temperature (300 K) using a VSM vibration frequency of 40 Hz and an amplitude of 2 mm. The sweep rate of the field was 100 Oe s^−1^.

**Table 1:** Characterization techniques for biominerals formed in the exterior and interior surface of biochar-treated root at micron- and sub-micron scale.

Techniques	Lateral resolution	Sample preparation	Characteristics of biominerals	Results
VSM magnetometry	n.a.	Critical point drying (CPD)	Magnetic nanoparticles with diameters of less than 50 nm in the root	Fig. S1
Raman microspectroscopy	~ 1 μm	CPD and longitudinal cutting	Micron-sized biochar particles on the root surface	Fig. S1
LA-ICP-MS	n.a.	Freeze drying and longitudinal cutting	The plant nutrients (i.e., Fe, Si, and P) significantly increased inside the biochar-treated root	Figure 1
ToF-SIMS	~ 100 nm	Freeze drying and longitudinal cutting	The iron oxides and hydroxides in the plaque layer	Figures 2, 3, and 4
SEM-EDX	~ 1 μm	CPD and longitudinal cutting and coating with chromium	Co-location of Fe- and Si-bearing ions at the root exterior and interior	Figures 3 and 4
STEM-EDX/EELS	< 0.14 nm	Embedding in Spurr’s resin and transverse cutting by ultramicrotomy (thickness ~ 60 nm)	(1) Nanoscale Fe–Si–P association on the root surface (2) ~ 5 nm iron core of ferritin inside the root (3) Intracellular needle-like clusters of iron nanoparticles in the aegirine phase	Figure 5 and Fig. S2
AFM	~ 100 nm	Embedding in Spurr’s resin and transverse cutting by microtomy (thickness ~ 400 nm)	Micron-sized electrically charged domains inside the root	Figure 6

### Raman microspectroscopy

The 1 cm long root samples after CPD were cut longitudinally and used for analysis. To identify the principal carbonaceous particles attached to the exterior surface of the root, Raman spectroscopy was performed using an inVia Raman microspectrometer (Renishaw, UK) using a 532 nm excitation source. Raman imaging was obtained using the StreamHR mode, over a 100 × 100 µm area, with a 20 × objective, 10% laser power (approximately 3.4 mW) with a 1 s exposure and a 500 nm step size. A map of the biochar particles’ Raman band at 1580 cm^−1^ on the root surface was collected using the WiRE software (Renishaw, UK).

### Laser ablation inductively coupled plasma mass spectrometry (LA-ICP-MS) and time-of-flight secondary ionization mass spectrometry (ToF-SIMS)

The longitudinal root sections were prepared as described above. The total concentrations of macronutrients (i.e., P, K, Ca, Mg, and Fe) and micronutrients (i.e., Mn, B, and Zn), as well as Si, Na, Al, Sr, and Ba at the exterior and interior surfaces of the root section were determined by LA-ICP-MS. The analysis was applied to randomly spots on each root using a NWR213 Laser Ablation unit (ESI New Wave, USA) coupled to a NexION 300D ICP-MS (Perkin Elmer, USA). Laser ablation parameters were as follows: wavelength 213 nm, repetition frequency (RF) 10 Hz, laser energy density 4.8 J cm^−2^ (at 30%), spot size 110 μm, depth 1.5 μm and scan speed 20 μm s^−1^. ICP-MS was performed at an RF power of 1150 W, helium gas flow rate of 0.8 L min^−1^, argon gas flow rate of 0.6 L min^−1^ in peak hopping scan mode and with a dwell time of 0.05 s. Elemental concentrations (calibrated against NIST610 and NIST612 glass standards) of the exterior and interior surfaces of the root were obtained in three separate 2 mm line scans for each area.

The molecular composition and distribution of the root interior and exterior on a sub-micron scale were measured using a ToF-SIMS 5 instrument (IONTOF GmbH, Germany) equipped with a bismuth liquid metal cluster ion gun as the primary ion source for analysis and an electron flood gun for charge compensation. Analysis was conducted using a 30 keV Bi_3_^+^ cluster ion beam on root sections mounted onto silicon wafers. Data acquisition was performed over regions ranging in area between 100 × 100 and 200 × 200 μm^2^ to generate (1) elemental and molecular information in the form of an accummulated mass spectrum, and (2) two-dimensional images showing the intensity distribution of selected secondary ions from the areas analysed. ‘Spectrometry’ mode was used to acquire high-mass resolution spectra (*m*/Δ*m* > 4000) for compositional analysis, and ‘fast imaging’ mode was used to acquire high spatial resolution images (lateral resolution ~ 200 nm, *m*/Δ*m* ∼ 200). To be consistent, both positive and negative spectra and images were collected from the same areas, resulting in four sets of data for each area analysed. Care was taken to ensure the total ion dose density from the four sets of scans was within the static SIMS limit (10^12^ primary ions per cm^2^). High-mass resolution positive spectra were calibrated using the masses of CH_2_^+^, C_2_H_4_^+^, C_4_H_8_^+^, and C_6_H_12_^+^ molecules. High-mass resolution negative spectra were calibrated using the masses of C_2_^−^, C_3_^−^, C_4_^−^, C_5_^−^, and C_6_^−^ molecules. The relative ion concentration was measured in three different locations for each sample and was normalised to the total ion count. The data processing and evaluation were conducted using the SurfaceLab 6 software package (IONTOF GmbH, Germany).

### Scanning electron microscopy coupled with energy-dispersive spectroscopy (SEM-EDS)

The longitudinal root sections were sputter-coated with chromium prior to analysis to make them electrically conductive. The morphology of the exterior and interior surface of the root was determined by SEM analysis. Data were obtained using the following SEMs: FEI Nova Nano SEM 230 and FEI 450 field-emission SEM (FEI, USA), each configured with a Bruker silicon drift detector energy dispersive X-ray spectrometer (EDS) (Bruker, USA). Elemental mapping, provided by EDS, was used to visualize the elemantal distributions in support of the other analytical methods. The spatial resolution of the EDS scans was ~ 6 μm.

### Scanning transmission electron microscopy coupled with energy-dispersive spectroscopy or electron energy-loss spectroscopy analyses (STEM-EDS/EELS)

Fresh roots were washed twice with deionized water and a transverse section of the root (~ 3 mm) was cut. Roots were fixed with 2.5% (v/v) glutaraldehyde PBS for one hour and then PBS alone was used to exchange the solution. Fixed tissues were washed three times with sodium cacodylate buffer and transverse sections were cut using a razor blade. Plant segments were post-fixed in 1% (w/v) osmium tetroxide in a sodium cacodylate buffer. The fixed samples were dehydrated in an increasing ethanol:water series (30%, 50%, 70%, 80%, 90%, and 100% v/v) and embedded in Spurr’s resin. During fixation, dehydration, and infiltration, Biowave microwave (Pelco, Redding, CA, USA) was used to speed up the rate of diffusion. Ultrathin sections of the root (~ 60 nm) were cut with a 45° diamond knife (Diatome, Biel, Switzerland) using an ultramicrotome (Leica EM UC6, Germany). Ultrathin sections were mounted on a lacey carbon support film on a 200 mesh copper support grid and stained with uranyl acetate (2%) and lead citrate.

STEM-EDS/EELS analysis was carried out using a ARM200F aberration-corrected STEM (JEOL, Japan) to investigate the ultrastructure of the root specimens. Stained specimens were imaged using bright-field modes at a 200 kV accelerating voltage in a scanning mode using a 8C (30 pA) probe and a 40 μm condenser aperture. The composition and speciation of compounds at sub-micron scale in the root were also determined. Compositional spectra at the regions of interest were analyzed by a NORAN System Seven X-ray Microanalysis System (Thermo Fisher, USA) coupled with a large area (1 sr) JEOL EDS detector. EEL spectra were recorded using a GIF model 963 Quantum Energy Filter (Gatan, USA) for determination of the oxidation state. Characterization of iron oxide nanoparticles in roots can be difficult using EELS, as many of the species are damaged or volatilized by the high-energy electron beam and the resin is readily damaged by the very high local electron intensity in the STEM probe. In addition, hydrocarbons on the specimen surface can diffuse to the beam location. Here they are cracked and carbonaceous deposits build-up, obscuring the details under study. To reduce these effects, STEM specimens were cooled to liquid nitrogen temperature using an in situ cooling sample holder prior to examination^54^.

### Atomic force microscopy (AFM)

The section (~ 400 nm) of resin-embedded root was cut using an ultramicrotome and a diamond knife. The surface topography and the distribution of the surface potential across a transverse section of root was determined using AFM (Dimension ICON, Bruker, USA). Tapping mode combined with lift mode was used to measure the surface topography and potential simultaneously (Kelvin probe force microscopy, KPFM). Firstly, the root section was placed onto a polished silicon wafer, the wafer was pre-fixed to an aluminium holder using silver paint. The sample was then placed and fixed onto the AFM stage. A cobalt-chromium coated AFM probe (SCM-PIT-V2, Bruker AFM probes) was installed onto the scanner head and used for all KPFM measurements. The probe was aligned and tuned near its resonance frequency with a slight offset. The lift height was kept to approximately 100 nm to avoid any topography artefacts. The scan area was set to 10 × 10 μm with a scan rate of around 0.6 Hz. The applied AC bias (drive2 amplitude) on the lift pass was set to 500 mV. Other parameters, such as setpoint, feedback gain and lock-in phase angle were optimized accordingly.

## Supplementary Information

Supplementary Figures.
