# Supplementary material for: Advanced characterization of biomineralization at plaque layer and inside rice roots amended with iron- and silica-enhanced biochar
Source: Sci Rep. 2021 Jan 8;11:159. doi: 10.1038/s41598-020-80377-z (PMC7794488; doi:10.1038/s41598-020-80377-z)
Supplement: Supplementary file 1 — Supplementary Figures. [file 41598_2020_80377_MOESM1_ESM.docx]

*Supplementary Information*

**Advanced characterization of biomineralization at plaque layer and inside rice roots amended with iron- and silica-enhanced biochar**

Guanhong Chen^1*^, Sarasadat Taherymoosavi^2^, Soshan Cheong^3^, Yao Yin^3^, Rabeya Akter^3^, Christopher E. Marjo^3^, Anne M. Rich^3^, David R.G. Mitchell^4^, Xiaorong Fan^5^, Jinkiat Chew^5^, Genxing Pan^5^, Lianqing Li^5^, Rongjun Bian^5^, Joseph Horvat^6^, Mohanad Mohammed^6^, Paul Munroe^2^, Stephen Joseph^2,6**^

^1^*National-Regional Joint Engineering Research Center for Soil Pollution Control and Remediation in South China, Guangdong Key Laboratory of Integrated Agro-environmental Pollution Control and Management, Institute of Eco-environmental and Soil Sciences, Guangdong Academy of Sciences, Guangzhou 510650, China.*

^2^*School of Materials Science and Engineering, UNSW, Sydney, NSW 2052, Australia.*

^3^*Mark Wainwright Analytical Centre, UNSW, Sydney, NSW 2052, Australia.*

^4^*Electron Microscopy Centre, AIIM Building, Innovation Campus, University of Wollongong, North Wollongong, NSW 2517, Australia.*

^5^*College of Resources and Environmental Sciences. Nanjing Agricultural University, Nanjing 210095-China*.

^6^*Institute for Superconducting and Electronic Materials and School of Physics, University of Wollongong, NSW 2522, Australia.*

***Corresponding author.** Tel.: +86 (0)20 87024633

****Corresponding author.** Tel.: +61 423629930

E-mail addresses: [ghchen@soil.gd.cn](mailto:ghchen@soil.gd.cn) (G. Chen);

[joey.stephen@gmail.com](mailto:joey.stephen@gmail.com) (S. Joseph)


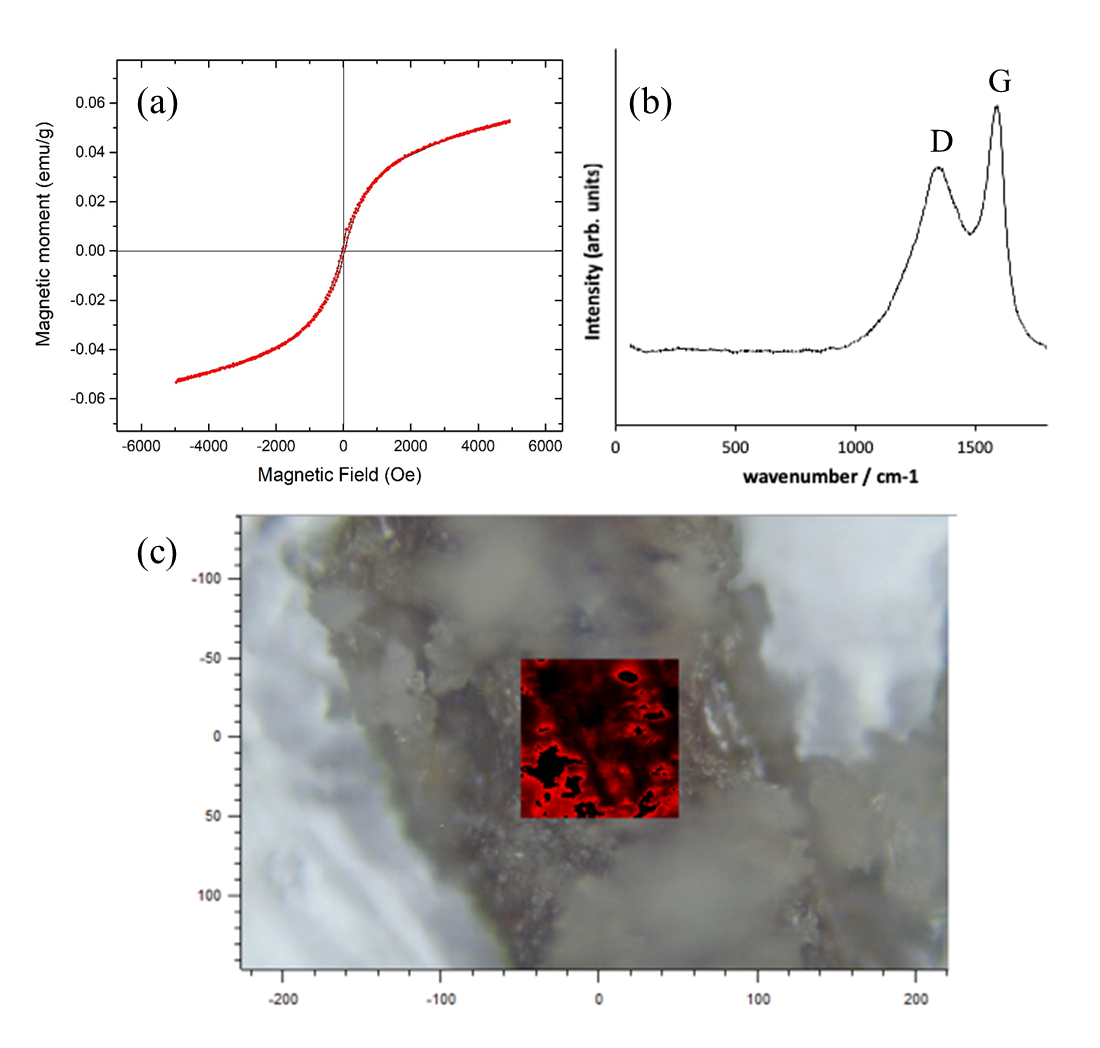


Figure S1. (a) Magnetization hysteresis curves measured at 298 K for the whole rice root sample. (b) Raman spectrum showing two overlapping bands at 1350 and 1580 cm^-1^. (c) Raman mapping of the G band is shown inset where micron-sized biochar particles appeared to form aggregates.


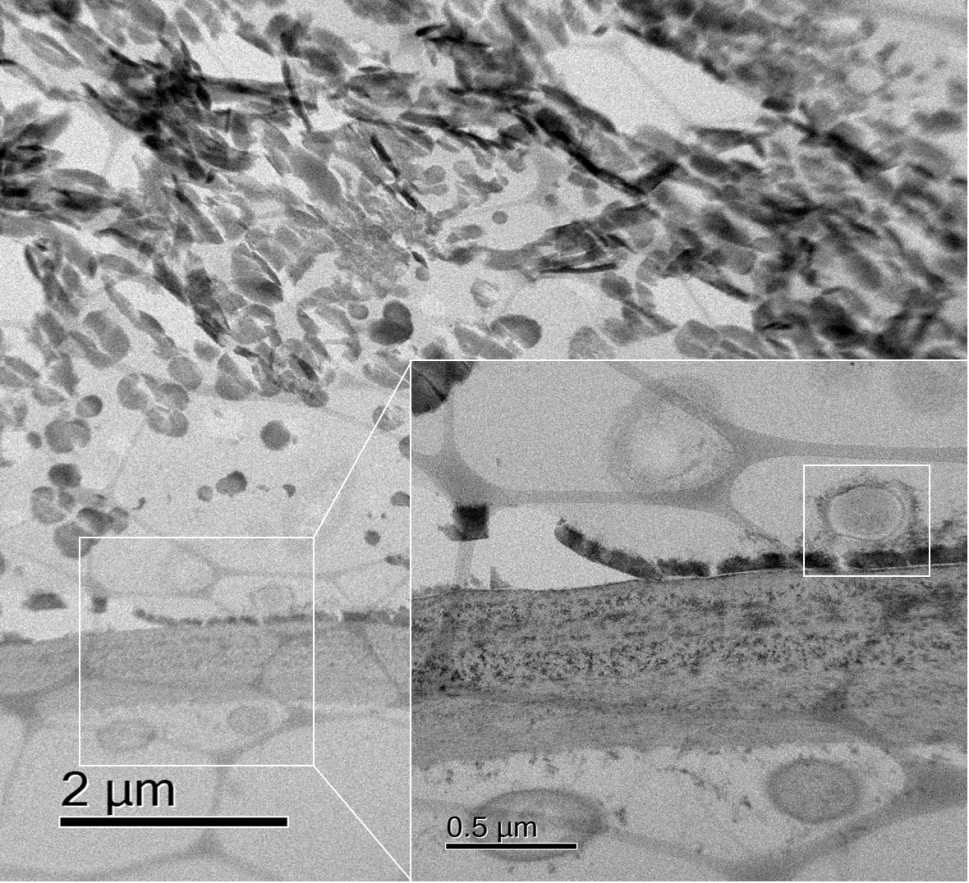


Figure S2. STEM analyses of a transverse section of a rice root exposed to enhanced biochar. Bright field (BF) image of a microbe attached to the root epidermis, and the magnified image of the microbe was shown in Fig. 5a.
